# The prescription of beta-blockers in older patients with heart failure with reduced ejection fraction: an observational study in Vietnam

**DOI:** 10.1038/s41598-024-63479-w

**Published:** 2024-06-05

**Authors:** Tan Van Nguyen, Hoa T. K. Nguyen, Wei Jin Wong, Fahed Ahmad, Tu Ngoc Nguyen

**Affiliations:** 1 Department of Geriatrics and Gerontology, University of Medicine and Pharmacy at Ho Chi Minh City, Ho Chi Minh City, Vietnam; 2 Department of Interventional Cardiology, Thong Nhat Hospital, Ho Chi Minh City, Vietnam; 3 Sydney School of Public Health, Faculty of Medicine and Health, The University of Sydney, Sydney, NSW Australia; 4 The George Institute of Global Health, UNSW, Sydney, Australia

**Keywords:** Cardiology, Epidemiology

## Abstract

This study in older hospitalized patients with heart failure with reduced ejection fraction (HFrEF) aimed to examine the prevalence of beta-blocker prescription and its associated factors. A total of 190 participants were recruited from July 2019 to July 2020. The inclusion criteria included: (1) aged ≥ 60 years, (2) having a diagnosis of chronic HFrEF in the medical records, (3) hospitalized for at least 48 h. The participants had a mean age of 75.5 ± 9.1, and 46.8% were female. Of these, 55.3% were prescribed beta-blockers during admission. To explore the factors associated with beta-blocker prescription, multivariable logistic regression analysis was applied and the results were presented as odds ratios (OR) and 95% confidence intervals (CI). On multivariate logistic regression models, higher NYHA classes (OR 0.49, 95%CI 0.26–0.94), chronic obstructive pulmonary disease (OR 0.17, 95% CI 0.04–0.85), chronic kidney disease (OR 0.40, 95% CI 0.19–0.83), and heart rate under 65 (OR 0.34, 95% CI 0.12–0.98) were associated with a reduced likelihood of prescription. In this study, we found a low rate of beta-blocker prescriptions, with only around half of the participants being prescribed beta-blockers. Further studies are needed to examine the reasons for the under-prescription of beta-blockers, and to evaluate the long-term benefits of beta-blockers in elderly patients with HFrEF in this population.

## Introduction

Heart failure in older people is a growing and evolving public health challenge, contributing to added pressures on healthcare systems worldwide^1,2^. Heart failure is a clinical syndrome characterized by symptoms and signs resulting from any structural or functional impairment of ventricular filling or ejection of blood^2,3^. The prevalence and incidence of heart failure increase significantly with age and can reach approximately 20% in people 75 years and older^4^.

Apart from age being an irreversible significant risk factor, other geriatric factors such as multimorbidity, polypharmacy, cognitive impairment and frailty can make the management of heart failure in older people complex^1,5^. With the growing population of older people and how the prevalence of heart failure doubles with each decade, managing heart failure in older people continues to be one of the major challenges in cardiovascular care^2,6^.

The major goals of therapy in heart failure management include improving quality of life, reducing mortality risk and preventing hospital readmission^2,7^. Studies have shown that in patients with heart failure, particularly heart failure with reduced ejection fraction (HFrEF), treatment with beta-blockers can reduce mortality and hospitalization risk, and improve their clinical status, symptoms of heart failure and left ventricular ejection function^2,8–13^. The benefits of beta blockers were consistent in older patients^3,14,15^. In patients with HFrEF, long-term treatment of beta-blockers is recommended to reduce the risk of major cardiovascular events^2,3^. However, beta blockers are sometimes underused in practice for various reasons, particularly in older patients with HFrEF^16^.

In Vietnam, heart failure was responsible for approximately 15% of all hospitalizations^17,18^. Older patients with heart failure in Vietnam had a high burden of symptoms which may require more resources for care, adding further strain to the health system^19^. However, there is limited evidence of the pharmacological treatment of heart failure in older adults, particularly the use of beta blockers in this population. Against this context, there is a need for more studies to better understand how care is provided for older patients with heart failure in Vietnam.

This study aims to examine the prevalence of beta-blocker prescription and its associated factors in older hospitalized patients with HFrEF in Vietnam.

## Methods

### Study design and population

This is a prospective observational study conducted at the Department of Interventional Cardiology and Department of Cardiology, Thong Nhat Hospital in Ho Chi Minh City, Vietnam from July 2019 to July 2020. Patient selection was carried out by reviewing the medical records. Patients were invited to participate in the study if they met the following criteria: (1) aged ≥ 60 years, (2) having a diagnosis of chronic HFrEF in the medical records, (3) hospitalized for at least 48 h. Patients were excluded from this study if (1) they had de novo heart failure; (2) they had contra-indications of using beta-blockers (such as cardiogenic shock, 2nd or 3rd degree AV block, sick sinus syndrome, liver failure, severe chronic pulmonary obstructive disease); (3) they did not provide consent; (4) they died during hospitalization.

All consecutive patients admitted to the hospital with heart failure were invited to participate in the study. If a patient admitted to the hospital more than once during the study period, the first admission that had a length of hospitalization > 48 h was chosen.

### Ethics approval and informed consent

The study was approved by the Ethics Committee of Thong Nhat Hospital in Ho Chi Minh City, Vietnam (107/2020/BVTN-HDYD). Informed consent was obtained from all participants. This study was compiled in accordance with the Declaration of Helsinki.

### Data collection

Data were collected from medical records. Information obtained included: demographic characteristics, height, weight, heart rate and blood pressure measures, comorbidities, medications prescribed during admission, and laboratory results during admission.

#### Outcome definition

The outcome of this study was the prescription of evidence-based beta-blockers for the treatment of HFrEF (including bisoprolol, carvedilol, metoprolol, nebivolol). Beta-blocker prescription was defined based on the medical records during admission.

#### Predictive variables

The potential variables that can be associated with the prescription of beta-blockers included age, sex, body mass index, New York Heart Association (NYHA) classes, heart rate, comorbidities including hypertension, ischemic heart disease, atrial fibrillation, valvular heart disease, dilated cardiomyopathy, diabetes, dyslipidemia, chronic obstructive pulmonary disease, and chronic kidney disease.

### Statistical analysis

Analysis of the data was performed using SPSS for Windows 29.0 (IBM Corp., Armonk, NY, USA). Continuous variables are presented as means ± standard deviation, and categorical variables as frequencies and percentages. Comparisons between groups (with and without beta-blocker prescriptions) were conducted using the Chi-square test or Fisher’s exact test for categorical variables and Student’s t-test or Mann–Whitney test for continuous variables.

To explore the factors associated with beta-blocker prescription, multivariable logistic regression analysis was applied. First, univariable logistic regression was performed on all the potential associated factors in the dataset, based on clinical rationale (such as age, sex, comorbidities, NYHA classes, and comorbidities). Variables that had a p-value < 0.05 on univariable analysis were selected for multivariable analysis.

Results were presented as odds ratios (OR) and 95% confidence intervals (CI).

## Results

### Participants characteristics

A total of 190 participants were recruited. Their mean age was 75.50 (± 9.05) years and 46.8% of the participants were female. NYHA III was the most common heart failure severity stage, occurring in 66.8% of patients. The average number of comorbidities were 3 (± 1.16), with the most common comorbidity being hypertension (83.7%), followed by ischemic heart disease (80.5%), diabetes mellitus (35.3%), dyslipidemia (34.7%), and atrial fibrillation (31.6%) (Table 1).

**Table 1:** General characteristics.

Characteristics	All participants (n = 190)	Participants with beta-blocker prescription (n = 105)	Participants without beta-blocker prescription (n = 85)	p value
Age (years)	75.50 ± 9.05	73.92 ± 8.87	77.45 ± 8.93	0.007
Sex				
Male	101 (53.2%)	50 (47.6%)	51 (60%)	0.089
Female	89 (46.8%)	55 (52.4%)	34 (40%)	
Smoking	12 (6.3%)	7 (6.7%)	5 (5.9%)	0.825
Body mass index				
Normal	96 (50.5%)	56 (53.3%)	40 (47.1%)	0.606
Underweight	33 (17.4%)	15 (14.3%)	18 (21.2%)	
Overweight	32 (16.8%)	17 (16.2%)	15 (17.6%)	
Obese	29 (15.3%)	17 (16.2%)	12 (14.1%)	
NYHA classes on admission				
NYHA I	0	0	0	0.287
NYHA II	61 (32.1%)	34 (32.4%)	27 (31.8%)	
NYHA III	127 (66.8%)	71 (67.6%)	56 (65.9%)	
NYHA IV	2 (1.1%)	0 (0%)	2 (2.4%)	
Hypertension	159 (83.7%)	91 (86.7%)	68 (80%)	0.216
Ischemic heart disease	153 (80.5%)	88 (83.8%)	65 (76.5%)	0.204
Diabetes	67 (35.3%)	37 (35.2%)	30 (35.3%)	0.994
Dyslipidemia	66 (34.7%)	38 (36.2%)	28 (32.9%)	0.640
Atrial fibrillation	60 (31.6%)	31 (29.5%)	29 (34.1%)	0.498
Chronic kidney disease	47 (24.7%)	16 (15.2%)	31 (36.5%)	< 0.001
Valvular heart disease	18 (9.5%)	11 (10.5%)	7 (8.2%)	0.600
Chronic obstructive pulmonary disease	13 (6.8%)	2 (1.9%)	11 (12.9%)	0.003
Dilated Cardiomyopathy	9 (4.7%)	4 (3.8%)	5 (5.9%)	0.504
Heart rate on admission (beat per minute)	84.65 ± 3.61	86.27 ± 15.74	82.65 ± 17.60	0.137
Systolic blood pressure on admission (mmHg)	126.44 ± 12.81	125.81 ± 16.83	127.22 ± 19.02	0.588
Diastolic blood pressure on admission (mmHg)	75.76 ± 9.77	75.47 ± 9.10	76.12 ± 10.59	0.649
Serum NT-proBNP level on admission (pg/mL)	5270.72 ± 7723.46	3669.45 ± 5113.77	7248.76 ± 9730.11	0.003
Serum creatinine on admission (mcmol/l)	129.85 ± 61.33	118.15 ± 60.84	144.37 ± 59.13	0.003
eGFR on admission (mL/min/1.73m^2^)	52.44 ± 19. 77	57.17 ± 18.62	46.60 ± 19.70	< 0.001
PAPs on admission (mmHg)	33.82 ± 14.25	32.76 ± 13.95	35.13 ± 14.58	0.256
LVEF on admission (%)	50.74 ± 15.06	50.44 ± 13.83	51.12 ± 16.53	0.758

NYHA, New York Heart Association; NT-proBNP, N-terminal pro b-type natriuretic peptide; eGFR, estimated glomerular filtration rate; PAPs, pulmonary artery pressure; LVEF, Left ventricular ejection fraction. Continuous variables are presented as means ± standard deviation, and categorical variables as frequencies and percentages.

**Figure 1:**
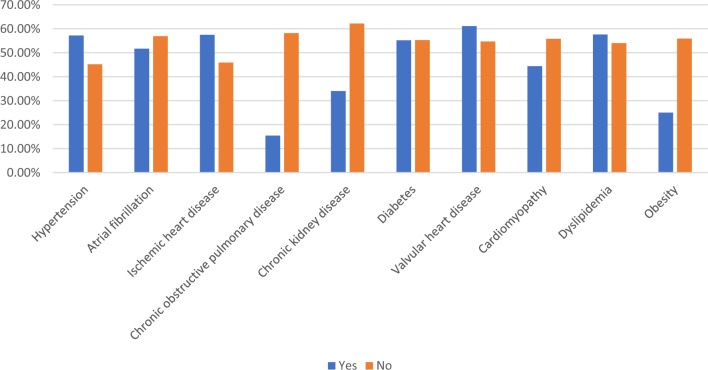
Prescription rates of beta-blockers by comorbidity types.

Among the 190 participants, 55.3% (105/190) were prescribed beta-blockers (83.8% received bisoprolol and 16.2% received nebivolol). Participants who did not receive beta-blocker prescription were significantly older (mean age 77.45 ± 8.93 versus 73.92 ± 8.87, p = 0.007), had higher prevalence of chronic kidney disease (36.5% versus 15.2%, p < 0.001), chronic obstructive pulmonary disease (12.9% versus 1.9%, p = 0.003), and had higher levels of NTproBNP (7248.76 ± 9730.11 versus 3669.45 ± 5113.77 pg/mL, p = 0.003) and serum creatinine (144.37 ± 59.13 versus 118.15 ± 60.84 mcmol/l, p = 0.003) compared to participants who were prescribed beta-blockers (Table 1). The prescription rates of beta-blockers by comorbidity types are presented in Fig. 1.

### Prescriptions of other medications for heart failure

Overall, 80.5% of participants were prescribed a renin-angiotensin system inhibitor (angiotensin-converting enzyme inhibitors—ACEIs, or angiotensin receptor blockers—ARBs), 68.9% received mineralocorticoid receptor antagonists (MRAs), 76.8% received loop diuretics, 6.3% received ivabradine, 26.3% received digoxin and 50.5% received nitrate.

Participants with beta-blocker prescription had significantly higher prescription rates of ACEIs/ARBs (89.5% versus 69.4%, p < 0.001), MRAs (75.2% versus 61.2%, p = 0.037), but lower rates of prescriptions of loop diuretics (67.6% versus 88.2%, p < 0.001) and nitrate (41.9% versus 61.2%, p = 0.008) compared to participants without beta-blocker prescription (Table 2).

**Table 2:** Prescriptions of other heart failure medications.

Medications	All participants (n = 190)	Participants with beta-blocker prescription (n = 105)	Participants without beta-blocker prescription (n = 85)	p value
ACEIs/ARBs	153 (80.5%)	94 (89.5%)	59 (69.4%)	< 0.001
MRAs	131 (68.9%)	79 (75.2%)	52 (61.2%)	0.037
Loop diuretics	146 (76.8%)	71 (67.6%)	75 (88.2%)	< 0.001
Ivabradine	12 (6.3%)	7 (6.7%)	5 (5.9%)	0.825
Digoxin	50 (26.3%)	30 (28.6%)	20 (23.5%)	0.433
Nitrate	96 (50.5%)	44 (41.9%)	52 (61.2%)	0.008

Categorical variables are presented as frequencies and percentages. ACEIs: angiotensin-converting enzyme inhibitors, ARBs: angiotensin receptor blockers, MRAs: mineralocorticoid receptor antagonists

### Factors associated with *beta*-blocker prescriptions

**Figure 2:**
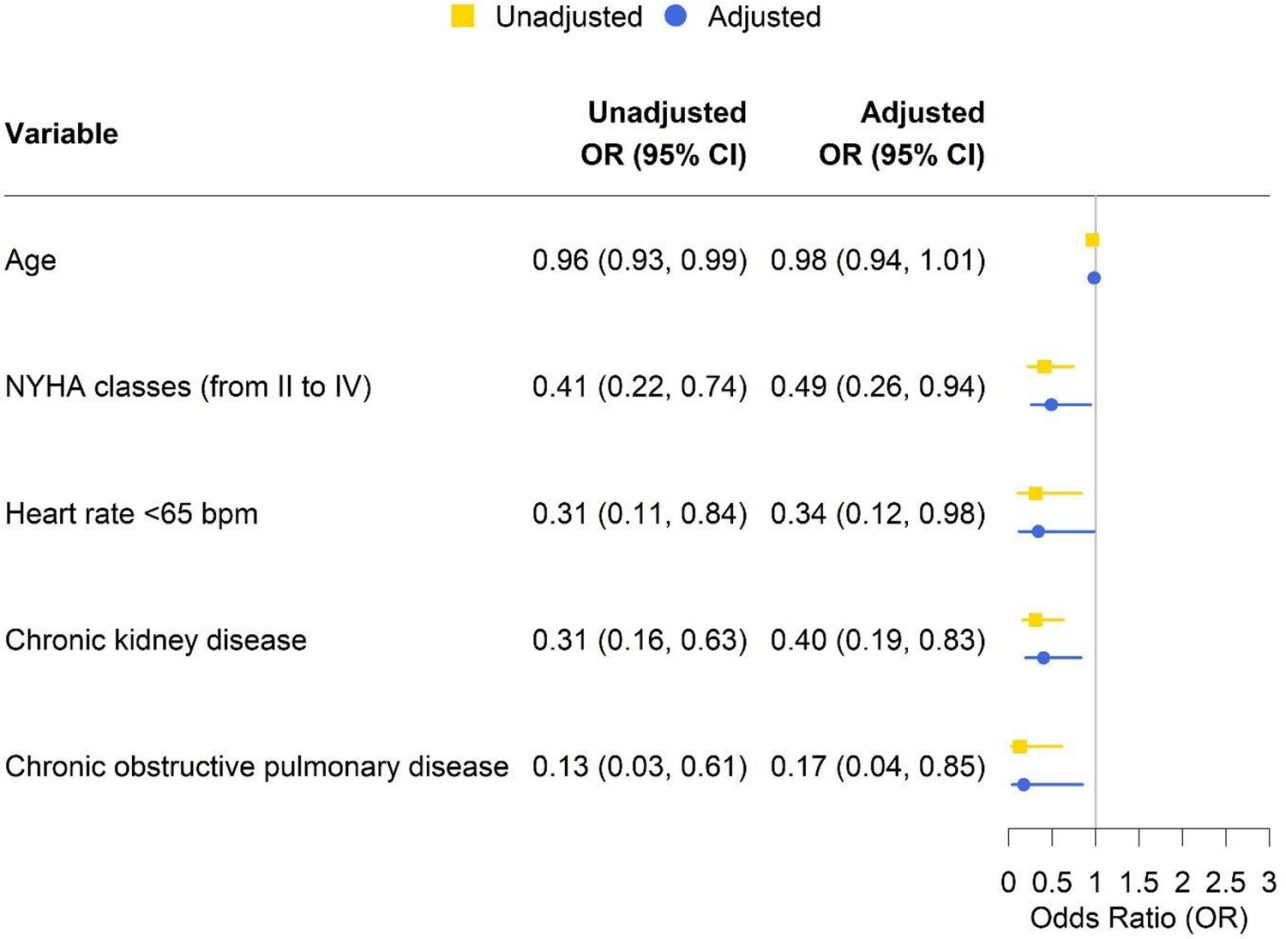
Factors associated with beta-blocker prescription in the adjusted model.

Table 3 describes the unadjusted odds ratios of the potential factors that can be associated with beta-blocker prescription. On multivariate logistic regression models, higher NYHA classes (OR 0.49, 95%CI 0.26–0.94), chronic obstructive pulmonary disease (adjusted OR 0.17, 95% CI 0.04–0.85), chronic kidney disease (adjusted OR 0.40, 95% CI 0.19–0.83), and heart rate < 65 bpm (adjusted OR 0.34, 95% CI 0.12–0.98) were significantly associated with a reduced likelihood of beta-blocker prescription (Fig. 2).

**Table 3:** Univariable logistic regressions of potentially associated factors for beta-blocker prescription.

Variables	Unadjusted odds ratios	95% confidence intervals	p
Age	0.96	0.93–0.99	0.008
Sex (female vs. male)	1.65	0.93–2.94	0.090
NYHA classes (from II to IV)	0.41	0.22–0.74	0.003
Heart rate < 65 bpm	0.31	0.11–0.84	0.021
Hypertension	1.63	0.75–3.52	0.219
Ischemic heart disease	1.59	0.77–3.28	0.206
Atrial fibrillation	0.81	0.44–1.50	0.498
Diabetes	0.99	0.55–1.82	0.994
Dyslipidemia	1.16	0.63–2.11	0.640
Valvular heart disease	1.30	0.48–3.52	0.601
Dilated cardiomyopathy	0.63	0.17–2.44	0.507
Chronic kidney disease	0.31	0.16–0.63	0.001
Chronic obstructive pulmonary disease	0.13	0.03–0.61	0.009
Obesity (body mass index ≥ 30)	0.26	0.03–2.57	0.251

## Discussion

In this study of 190 older hospitalized patients with HFrEF, there was low uptake of beta-blocker prescriptions, with only 55.2% of the participants prescribed beta-blockers. Participants with chronic obstructive pulmonary disease, chronic kidney disease, higher NYHA classes and heart rate < 65 bpm had lower odds of being prescribed beta-blockers.

Under-utilization of beta-blockers in patients with HErEF has been reported in many studies in other countries. In a study of 1825 patients (mean age 77.3 ± 13.2) admitted for heart failure for the first time between 2009 and 2011 in France, the prescription rates of beta-blockers before admission ranged from 26 to 32%, and 46–54% at discharge^20^. In a study of 1075 patients with heart failure (age range 62–84) in Central Asia and Europe in the period 2013–2019, the rates of beta-blocker use at admission in patients with HFrEF ranged from 43.5% in Central Asia to 59.1% in Europe^21^. In a study of 1509 patients (mean age 63.9 ± 16.1) hospitalized for acute HFrEF in 21 hospitals in Taiwan in 2014, 60% were prescribed beta-blockers at discharge^22^. Some other studies reported higher prescription rates of beta-blockers for patients with HFrEF. For example, a multi-centered study in 1853 older patients (age range 61–77) in Germany conducted from 2009 to 2011 reported that 71.0% of the participants were treated with beta-blockers at admission and 89% at discharge^23^. In a study of 1003 patients (mean age 54.4 ± 15.0) recruited from 2016 to 2019 in China, the prescription rate of beta-blockers in hospitalized patients with HFrEF was 84.2%^24^. The younger age of the participants and the fact that patients with heart rates < 75 bpm were excluded from that study may explain a higher prescription rate compared to our study^24^.

The novelty of our study lies in its unique contribution to the evidence of the prescription of beta-blockers among older patients with HFrEF within the context of Vietnam. Our study was the first to be specifically designed to examine the use of beta-blockers in older patients with HFrEF in Vietnam. There is a scarcity of research in older patients with heart failure in Vietnam and most of them focused on the quality of life and symptom burden^19,25,26^. In a pilot study in 257 patients with heart failure (mean age 64 ± 15 years) examining the implementation of the Optimize Heart Failure Care Program for patients with heart failure in Vietnam, the investigators noticed a low rate of prescription of beta-blockers at time of hospital discharge (approximately 33%), which is consistent with our finding^26^. Given the rising prevalence of cardiovascular diseases, particularly heart failure in this country, the limited number of studies highlights a crucial gap in understanding and addressing this health concern. Our research stands among the few studies addressing this critical health issue in older adults in Vietnam. This focus on an understudied population and therapeutic approach highlights the innovative nature of our work and its potential to inform and improve clinical practices in the country. Our research provides valuable insights into the local epidemiology and management practices. The low prescription of beta-blockers raises concerns about inadequate management and the need for increased promotion and education on beta-blocker use in elderly patients with HFrEF in Vietnam. In this study, although all beta-blockers prescribed were β1-selective agents (bisoprolol and nebivolol), the presence of chronic obstructive pulmonary disease was significantly associated with reduced odds of receiving beta-blockers (by 83%). This may reflect the extra-caution of the physicians in prescribing beta-blockers for older patients with chronic obstructive pulmonary disease, although research has affirmed that selective β1-blockers are safe in patients with concomitant HFrEF and chronic obstructive pulmonary disease^16^. Of note, our study found that chronic kidney disease was presented in one-quarter of the studied participants and reduced the odds of receiving beta-blockers by 60%.

More studies are needed in older patients with HFrEF in Vietnam to understand the factors associated with the non-prescription of beta-blockers. The reasons for the low prescription of beta blockers among older patients with HFrEF are usually multi-faceted. Due to the higher comorbidities and increased risk of adverse side effects in older patients, physicians may be reluctant to prescribe beta-blockers. Polypharmacy and concomitant use of other medications are other aspects that can limit the prescription of beta-blockers. These results emphasize the need for continued vigilance towards the utilization of evidence-based treatment strategies, including evidence-based beta-blockers in older patients with HFrEF. The results of this study imply that healthcare providers need to assess their current prescribing practices of beta-blockers in older patients with HFrEF. By addressing the identified barriers to prescribing beta-blockers, healthcare providers can improve the management of HFrEF in older patients.

There are a few limitations to this study. Firstly, it was carried out on older patients from one hospital, so it may not accurately reflect all older patients with HFrEF in Vietnam. The inclusion criterion of having a diagnosis of chronic HFrEF was based on the medical records and we were unable to capture details such as the duration of having a diagnosis of HFrEF and the participants’ previous EF measurements, so there is a chance that several participants with HFpEF may be mis-classified as HFrEF. In addition, we did not collect information related to socioeconomic status, diet, or other local-specific factors and patient-specific factors (such as a history of intolerance and side effects of beta-blockers) that may have an influence on the prescription of beta-blockers. Therefore, the study findings should be cautiously interpreted.

In conclusion, in this study only around half of the participants with HFrEF were prescribed beta-blockers. These results suggest that the use of beta-blockers in older patients with HFrEF needs more attention to improve patient outcomes. Further studies are needed in older patients with HFrEF in Vietnam to examine the reasons for the under-prescription of beta-blockers, and to evaluate the long-term benefits of beta-blocker use in elderly patients with HFrEF.

## Data Availability

The dataset used and/or analyzed during the current study are available from the corresponding author on reasonable request.
